# An innovative approach to improve the detection and treatment of risk factors in poor urban settings: a feasibility study in Argentina

**DOI:** 10.1186/s12889-021-10569-3

**Published:** 2021-03-22

**Authors:** Poggio Rosana, Goodarz Danaei, Laura Gutierrez, Ana Cavallo, María Victoria Lopez, Vilma Irazola

**Affiliations:** 1 Department of Research in Chronic Diseases, Institute for Clinical Effectiveness and Health Policy (IECS), Emilio Ravignani 2024 (C1414CPV), Buenos Aires, Argentina; 2 Department of Global Health and Populations. Department of Epidemiology, Harvard T.H. Chan School of Public Health, Boston, MA USA

**Keywords:** Cardiovascular risk, Community health workers, Implementation research, Hypertension, Diabetes, Primary health care, Feasibility study

## Abstract

**Background:**

The effective management of cardiovascular (CVD) prevention among the population with exclusive public health coverage in Argentina is low since less than 30% of the individuals with predicted 10-year CVD risk ≥10% attend a clinical visit for CVD risk factors control in the primary care clinics (PCCs).

**Methods:**

We conducted a non-controlled feasibility study using a mixed methods approach to evaluate acceptability, adoption and fidelity of a multi-component intervention implemented in the public healthcare system. The eligibility criteria were having exclusive public health coverage, age ≥ 40 years, residence in the PCC’s catchment area and 10-year CVD risk ≥10%. The multi-component intervention addressed (1) system barriers through task shifting among the PCC’s staff, protected medical appointments slots and a new CVD form and (2) Provider barriers through training for primary care physicians and CHW and individual barriers through a home-based intervention delivered by community health workers (CHWs).

**Results:**

A total of 185 participants were included in the study. Of the total number of eligible participants, 82.2% attended at least one clinical visit for risk factor control. Physicians intensified drug treatment in 77% of participants with BP ≥140/90 mmHg and 79.5% of participants with diabetes, increased the proportion of participants treated according to GCP from 21 to 32.6% in hypertensive participants, 7.4 to 33.3% in high CVD risk and 1.4 to 8.7% in very high CVD risk groups. Mean systolic and diastolic blood pressure were lower at the end of follow up (156.9 to 145.4 mmHg and 92.9 to 88.9 mmHg, respectively) and control of hypertension (BP < 140/90 mmHg) increased from 20.3 to 35.5%.

**Conclusion:**

The proposed CHWs-led intervention was feasible and well accepted to improve the detection and treatment of risk factors in the poor population with exclusive public health coverage and with moderate or high CVD risk at the primary care setting in Argentina. Task sharing activities with CHWs did not only stimulate teamwork among PCC staff, but it also improved quality of care. This study showed that community health workers could have a more active role in the detection and clinical management of CVD risk factors in low-income communities.

**Supplementary Information:**

The online version contains supplementary material available at 10.1186/s12889-021-10569-3.

## Background

Argentina has a population of approximately 44 million people (91% in urban areas) [1] and has become the highest contributor to cardiovascular disease (CVD) burden in South America (32% of CVD deaths in the region). The principal CVD determinants are hypertension, high cholesterol, diabetes and obesity [2].

Slightly more than a third of the population (36%) are covered exclusively by the public health care system and this proportion is much higher among the poorest [1] households. When compared to the most affluent, this population showed a higher prevalence of hypertension (46 vs 37.5%), obesity (28.1 vs 17%), diabetes (14.6 vs 8.1%) and high cholesterol (42 vs 26.4%) [3]. Health care coverage for this population is provided by a free-access public health care system, consisting of primary care clinics (PCC) and hospitals.

To address the growing burden of CVD and health disparities, provincial and national ministries of health have implemented several programs with the objective of improving CVD prevention in the population with exclusive public health coverage at the primary care level.

For example, the “Red Pública de Salud AMBA” [4] is a comprehensive program to strengthen and improve the quality of the service provided by PCCs. The “SUMAR” [5] and “PROTEGER” [6] programs provide funds conditional on quality criteria for the detection, treatment, and follow-up of people with diabetes or high blood pressure, childhood obesity and funds for building healthy environments. The “REDES” [7] and “REMEDIAR” [8] programs provide training for health care providers to detect individuals with moderate or high CVD risk and supply free drugs (antihypertensive, hypoglycemic and lipid-lowering) for risk factor control in the PCCs.

Despite these efforts, less than 30% of the individuals at moderate to high CVD risk (i.e. predicted 10-year risk of ≥10%) attend the clinical visit for CVD risk factor control in the PCC [9–11] and risk factor control rates among individuals with hypertension, diabetes and high cholesterol are low (21, 40 and 11.1% respectively) [12–14]. Previous studies have shown the main barriers of effective risk factor management at individual level are low educational level, low health care literacy, and reluctance to take medication. At provider level, low adherence to clinical practice guidelines, predisposition to accept uncontrolled risk factors, and lack of time. At system level, issues arise mainly from difficulties to schedule appointments with primary care physicians, communication problems between primary health care staff, community health workers (CHW) activities being mainly focused on maternal and childcare at the PCCs [15, 16].

There is abundant evidence demonstrating the superiority of complex interventions in controlling risk factors compared to individual components [17–19]. Three cluster trials tested multi-component interventions to improve the management and control of risk factors among vulnerable population in Argentina [18, 20, 21]. Each study combined different proven effective components such us household approach led by CHWs, educational programs to physicians, mHealth applications (to provide medical appointments, 10-years CVD risk calculation) and sending text messages to participants with reminders or health education information.

The hypertension control trial was effective in reducing blood pressure (BP) levels (net difference − 4.8 mmHg in systolic and − 3.3 mmHg in diastolic BP) [18]. The study carried out on individuals with hypercholesterolemia increased the adequate use of clinical practice guidelines among physicians (net difference + 38.5%) with no effect on LDL [20]. The third study was implemented on the high CVD risk population and it reported increased attendance to the clinical visit at the PCCs for risk factor control (net difference + 36.7%); the mean number of visits in the intervention group during the study period (in 6 months) was 1.1 and had no effect on medical treatment of chronic conditions [21].

Attendance to follow up visits for risk factor control at the PCCs, adherence to clinical practice guidelines, predisposition to accept uncontrolled risk factors among providers, and communication problems among primary health care staff continue to be a challenge for future interventions aiming to improve risk factor control. Therefore, the overall aim of this study was to evaluate the feasibility of a new multi-component intervention integrating some individual components previously proven feasible and effective with the addition of other novel components among the poor population with exclusive public health coverage and with moderate or high CVD risk in Argentina.

## Methods

### Overview of the study design

The study was conducted in Marcos Paz, located in the province of Buenos Aires, Argentina [22]. The population is 54.181 inhabitants, mostly located in urban areas (80%) and 62% are users of the public health system, consisting in one hospital, 8 PCCs and 1 itinerant health care post.

**Fig. 1:**
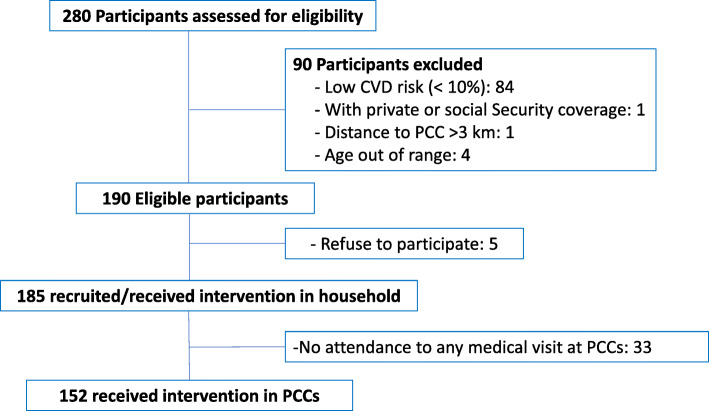
Flow Diagram of Study Participants

We implemented a non-controlled feasibility study using a mixed method approach to evaluate fidelity, adoption and acceptability of a multi-component intervention implemented in six PCCs. The eligibility criteria for study participants were having exclusive public health coverage, age ≥ 40 years, residence in the PCC’s catchment area, and 10-year CVD risk ≥10%. All participants had free access to antihypertensive, hypoglycemic, and lipid-lowering drugs. The intervention and follow-up lasted 6 months. The flow diagram of the study participants is described in Fig. 1.

### Screening and recruitment process

Trained and certified CHWs carried out an active search of individuals with 10-year CVD risk ≥10% in the catchment area of the PCCs, following the procedures used by the national program REDES. They visited the participants’ homes and estimated the 10-year CVD risk, using the CVD charts for risk stratification without cholesterol measurements developed by the World Health Organization for the Latin America and Caribbean [23]. In the screening/baseline visits, CHWs confirmed patients’ eligibility, and those who met the inclusion criteria were invited to participate in the study. After patients’ acceptance and consent, CHWs proceeded to deliver the intervention in the patient’s home. Subjects who did not meet the inclusion criteria received a short advice on the importance of risk factors control and were thanked for their time.

### Intervention

The multicomponent intervention program addressed system, provider, and individual barriers to risk factor detection and treatment by integrating some individual strategies previously proven effective with novel components (Table 1).

**Table 1:** Summary of the strategies to overcome barriers for risk factors evaluation and treatment

Barrier	General approach	Specific strategy to overcome barrier
1. System/ organizational Level		
-Multiple competitive demands on physicians’ time -Inadequate incentives for professionals to promote prevention actions -Community health workers activities mainly focus on maternal and childcare	Task shifting	●Simplify the physician’s task by assigning the CVD risk stratification and counseling for cardiovascular health care to CHWs
Difficulties in scheduling medical appointments in PCCs	Organizational change	● Creation of protected medical appointment slots
Communication problems in the interface among the primary health care staff	Team-based approach	● New CVD form that centralizes the clinical registry of physicians, nurses and CHWs
2. Provider Level		
-Low adherence to clinical practice guidelines -Predisposition to accept uncontrolled risk factors	Physician education Aid tools	● 1 Workshop session in the use of guidelines, treatment algorithms ● 2 Educational outreach visits: prescribing audit and feedback ● Pocket card with drug treatment as decision trees ● New CVD form with a special section to register drug treatment
3. Individual Level		
Lack of CV care knowledge risk perception	Family education	● Cardiovascular disease care counselling to participant and family in participant’s household
Poor attendance to the PCC	Appointment reminders, and Family support	● CHW will provide a card with the medical appointment record ● Family members help to remind each other
Low health literacy Reluctance to take medication	Patient education	● Counselling provided by CHWs, who are from the local community, to ensure that health information is culturally and linguistically appropriate ● Distribution of printed educational material
Lack of time	Organizational change	● Medical appointment provided in participant’s household

The intervention addressed system barriers through task shifting [17, 24–26] among the PCC’s staff by assigning CVD risk stratification, health care counseling, greater involvement in the follow up of risk factors to community health workers, and creation of protected medical appointment slots [24–26]. A new form was specially designed for this study (Figure S1). The novelty of this form is that it includes three new sections to be completed by physicians, nurses and CHWs, aiming to stimulate teamwork. The new CVD form was designed based on the current form used by the national program REDES with the addition of new sections: I) Risk factors status showing the results in traffic light signs to facilitate its visualization by physicians and other PCC’s staff and current drug treatment (to be completed by CHWs); II) Clinical measurements (BP values, weight, height and BMI calculation, to be completed by nurses) and III) Physician’s section (notes and prescription).

Provider barriers were addressed through educational outreach visits (EOVs) [27, 28]. The study protocol proposed that physicians attended two EOVs during the study period. Educational outreach visits have demonstrated to change health professionals’ practice, particularly the prescribing patterns of physicians [28]. Trained peer physicians conducted the EVOs in site, including the audit of clinical cases and feedback on prescription practices. In addition, physicians were provided with aid tools (pocket-cards summarizing the guidelines algorithms as decision-trees) to facilitate both pharmacological and non-pharmacological treatment [29].

Individual barriers through a home-based intervention delivered by CHWs [25, 30, 31]. They conducted home visits, in which they provided printed educational materials, conducted health care counselling sessions about to the importance of adhering to healthy lifestyles and taking medications to participants and their families using the traffic light signs of the new CVD form. Then CHWs scheduled a medical appointment from the protected medical appointment slots and provided participants and their families with printed educational materials and a card with the appointment record [32–36]. The study flow is summarized in Figure S2.

### Study outcomes

The primary outcomes were feasibility outcomes including acceptability, adoption and fidelity, expressed as the proportion of 1) CHWs and physicians who completed the training sessions. 2) Eligible individuals with the new CVD form completed by CHWs, nurses and physicians 3) Counselling sessions and medical appointment provided to participants. 4) Individuals who attended the scheduled physician’s appointment at the PCC. 5) Outreach visits successfully conducted, and 6) Participants who reported to be satisfied/very satisfied with the multi-component intervention.

The secondary outcomes were constituted by intermediate and effectiveness indicators. The intermediate indicators were: 1) Proportion of individuals with hypertension who received drug treatment according to Argentinian good clinical practice guidelines (GCP) [37]. 2) Proportion of individuals with diabetes treated with the first-line drug proposed by Argentinian GCP; and 3) Proportion of individuals who received statins and aspirin according to their estimated cardiovascular risk. The effectiveness indicators were: 4) Proportion of participants with BP < 140/90 mmHg and 5) Proportion of participants with fasting glucose < 126 mg/dL.

We define moderate CVD risk as individuals with estimated 10-years risk of 10 to 19%. High CVD risk as individuals with estimated 10-years risk of 20 to 29% and very high CVD risk as ≥30%. Hypertension was defined as participants with BP values ≥140/90 mmHg OR under drug treatment at baseline for hypertension control. Controlled BP was defined as participants with BP values < 140/90 mmHg. Diabetes was defined as participants with history of diabetes (self-reported) OR under drug treatment at baseline for diabetes control.

### Data collection

CHWs collected baseline data related to socioeconomic variables, medical history and use of drug treatment for risk factors control during the baseline visit in the participant’s household, using standardized questionnaires specially developed for this study. Independent personnel collected the study outcomes from administrative and medical records after 6 months of the enrollment visit. Qualitative data was collected with a convenient sampling to assess acceptability among providers (4 physicians and 6 CHWs) and satisfaction level in participants through a telephone interview. (Supplementary material: Study forms).

### Data analysis

For the quantitative analysis, we used descriptive statistics such as means, median and proportions to describe the general characteristics of the study population, study outcomes, process indicators and satisfaction level in participants using the RE-AIM framework [38]. Statistical analyses were performed using STATA version 12.0 (Stata Corp., College Station, TX, USA). For the qualitative analysis, written transcripts of the interviews were classified and then codified according to the study objectives. The written transcripts were entered into ATLAS.ti version 7 software (ATLAS.ti Scientific Software Development GmbH) combined with the manual technique of information coding. Analytical dimensions were identified as constructs for the description of findings. Finally, data were abstracted and interpreted through content analysis [39, 40].

## Results

The recruitment phase lasted 2.5 months. One hundred eighty-five participants received the intervention at home and 152 of those attended the clinic visit at the PCC (Fig. 1). The mean age was 57.3 years, constituted mostly by women (69%), low educational level (89% has primary school level or less), and low proportion of active workers (81% unemployed, retired or homemaker). (Table 2). Mean systolic and diastolic blood pressure values were 154.1 and 91.6 mmHg, respectively; 92.4% of participants had hypertension (high blood pressure or under treatment at baseline), 69% of which were under treatment; however, only 13.5% of total hypertensive participants had controlled BP values. Most hypertensive participants were taking only one drug (79%), enalapril being the most frequently prescribed drug. Diabetes prevalence was 41.1, and 75% were under treatment. The CVD risk estimate showed that 35% had moderate risk, 20% high and 45% very high-risk. The use of statins, according to CVD risk, was low in both high and very high-risk groups (11 and 7% respectively). The use of aspirin within the very-high risk group was also low (37%).

**Table 2:** Baseline Characteristics of the study population

	n 185
Age, mean (SD), y	57.3 (8.5)
Female sex, n (%)	127 (68.6)
Primary school or less, n (%)	164 (88.6)
Unemployed / Retired / Homemaker, n (%)	149 (80.5)
Household members, median (IR)	3.0 (2–5)
Currently smoking, n (%)	35 (18.9)
History of major CVD, n (%)	29 (15.7)
Systolic BP, mean (SD), mm Hg	154.1 (22.3)
Diastolic BP, mean (SD), mm Hg	91.6 (13.7)
Hypertension, n (%)^a^	171 (92.4)
Under treatment	118 (69.0)
Number of drugs prescribed for hypertension control	
1 Drug	93 (78.8)
2 Drugs	14 (11.9)
3 or more drugs	11 (9.3)
Controlled BP	23 (13.5)
Diabetes mellitus (history DM or treatment), n (%)^b^	76 (41.1)
Under treatment	56 (74.7)
Moderate CVD Risk (10–19%), n (%)	65 (35.1)
High CVD risk (20–29%), n (%)	37 (20.0)
Use of statins	4 (10.8)
Very high CVD risk (≥ 30%), n (%)	83 (44.9)
Use of statins and aspirin	6 (7.2)
Use of statins	6 (7.2)
Use of aspirin	28 (37.3)

*SD* standard deviation, *IR* interquartile range, *BP* blood pressure. ^a^Hypertension: participants with BP values ≥140/90 mmHg OR under drug treatment at baseline for hypertension control. ^b^Diabetes: History of diabetes (self-reported) OR under drug treatment at baseline for diabetes control

### Feasibility outcomes and process indicators

All CHWs (100%) and physicians (100%) attended the training session for primary care physicians. (Table 3). The counseling session was provided in 97.8% of recruited participants, and the most frequently delivered modules were CVD risk and nutritional. The median of household members who received the counseling session was 2, and CHWs provided the appointment for the clinical visit to 93.4% of participants. After the household visit, CVD forms were included in 93% of participant’s medical records and 97.7% of them had the CHWs’ sections completed (risk factors and drug treatment sections).

**Table 3:** Feasibility outcomes and process indicators

	n (%)
I. Training component	
CHWs with complete training session	12/12 (100)
Physicians with complete workshop	5/5 (100)
II. Household component	**n 185**
Counseling session provided	181 (97.8)
CVD risk module	162 (89.5)
Nutritional module	129 (71.3)
Physical activity module	89 (49.2)
Household members who received the counseling session, median (IR)	2.0 (1–2)
Medical appointment provided	169 (93.4)
CVD form included in medical records after household visit	172 (93.0)
Complete in CHW’s section	168 (97.7)
III. Clinic component	
a. Attendance to clinical visit	
Participants attending at least 1 clinical visit	152 (82.2)^a^
Number of clinical visits during the study period, mean (SD)	1.8 (0.9)
1 visit	68 (44.7)
2 visits	51 (33.6)
3 or more visits	33 (21.7)
b. CVD form	
Included in medical records during clinical visit	152 (100)
Physicians’ section complete^a^	143 (94.1)
Nurses’ section complete^a^	135 (88.8)
c. Clinical measurements registration by nurses	
BP	148 (97.4)
Height and weight	141 (92.7)
BMI calculation	135 (88.8)
d. EOVs and primary care physician’s practice	
EOVs successfully conducted	10/10 (100)
Fasting glucose requested in diabetic participants	44/64 (68.8)
Glycosylated hemoglobin requested in diabetic participants	12/64 (18.8)
Drug intensification in those with any glycemic parameter available	35/44 (79.5)
Drug intensification in those with BP ≥140/90 mmHg^b^	87/113 (77.0)
e. Participants satisfaction with the multi-component intervention^c^	99/103 (96.0)

^a^At least in one visit at PCC. ^b^Increased doses or drugs; *EOVs* educational outreach visit, *CHW* community health worker, *BP* blood pressure, *BMI* body mass index. ^c^Satisfied or very satisfied

Attendance to at least one clinical visit for risk factor control was 82.2%. The mean number of clinical visits during the study period was 1.8 (SD 0.9), 45% attended only one visit, 33.6% two visits, and 21.7% three or more visits. Physicians had 100% of the CVD forms available to carry out the evaluation in participants that attended at least one clinical visit for risk factor management at the PCC. At the end of follow up, 94.1% of CVD forms were correctly completed in the physicians’ sections (notes and drug prescription sections). The nurses’ section was correctly completed in 88.8% of forms (BP values, height, weight, and BMI calculation in at least one visit). Blood pressure was registered in 97.4%, height and weight 92.7%, and BMI in 88.8% of participants.

At the physician’s practice level, 100% of educational outreach visits proposed by protocol (2 per physician) were conducted. Among diabetic patients who attended at least one visit during follow up (n 64), physicians requested fasting glucose analysis in 68.8%, and glycosylated hemoglobin in 18.8%. Physicians increased doses or drugs for hypertension control in 77% of participants with BP ≥140/90 mmHg and 79.5% of participants with any glycemic parameter available.

Patients were highly satisfied with the intervention with 96% (99/103) responding that they were satisfied or very satisfied. The health promotion messages provided by CHW in the household visit were *totally unknown* for 61.2% of participants. Among those who attended at least one clinical visit (92), 89.1% used the medical appointment provided by CHW in the household visit. The most frequent reasons for not attending the clinical visits were due to “work reasons” (27.3%) and “forgot the appointment” (18.2%).

### Intermediate and effectiveness indicators

**Fig. 2:**
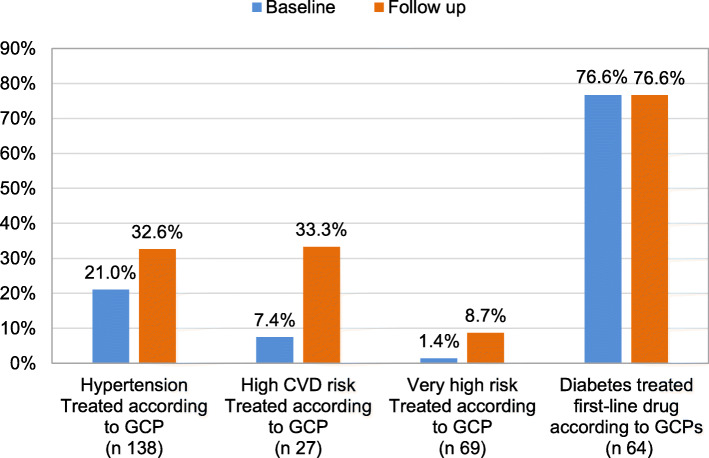
Participants treated according to guidelines at baseline and 6-month follow-up

When we compared baseline and follow up among intermediate outcomes in those with available data, we observed an increase in the proportion of participants treated according to GCP at the end of follow up (Fig. 2). In participants with hypertension (138), the proportion increased from 21 to 32.6%, high CVD risk (37) from 7.4 to 33.3% and very high CVD risk (83) from 1.4 to 8.7%. In participants with diabetes, there was no difference in the proportion of participants treated with the first-line drug (metformin) proposed by the Argentinians’ GCP guidelines between baseline and follow up, since it was 76.6% in both cases.

**Fig. 3:**
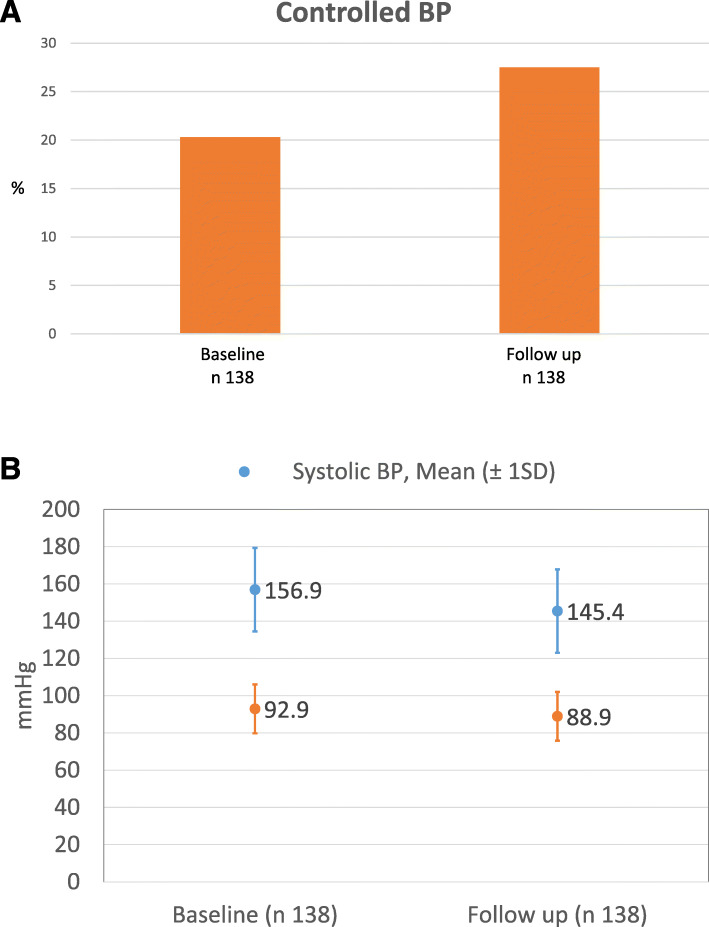
**a** Participants with hypertension and controlled BP at baseline and 6-month follow-up. **b**. Mean blood pressure values at baseline and 6-month follow-up

In those participants with hypertension and blood pressure measurements available attending at least 1 clinical visit (138), we observed an increase in the proportion of participants with controlled BP values (< 140/90 mmHg) at the end of the study from 20.3 to 27.5% (Fig. 3). When we compared blood pressure values, we also observed that mean systolic BP was 11.5 mmHg lower (from 156.9 to 145.4 mmHg) and diastolic BP 4 mmHg lower (from 92.8 to 88.9 mmHg) at the end of follow up. Among participants with diabetes and fasting glucose data available during follow up (*n* = 44), 31.8% (14) finished the study with controlled blood glucose levels (< 126 mg/dL).

### Qualitative results

CHWs and providers accepted the intervention fairly well and considered the strategy to be useful to detect more patients with uncontrolled risk factors in the community. They also reported that the intervention stimulated teamwork and improved communication among the PCC staff. CHWs showed great enthusiasm for having a more relevant role in the patient’s health care and commented that the new CVD form (showing risk factor status in traffic light signs) facilitated the interpretation of results to CHWs and was didactic to explain the results to participants. Similarly, physicians considered the addition of the new CVD form to medical records valuable since it helped to better organize and improve the quality of their own work. Physicians commented that educational outreach visits helped them to “improve/ change” their prescription patterns, especially in prescribing statins and aspirin according to CVD risk.

## Discussion

Our results showed that the proposed CHWs-led intervention was feasible and well accepted in improving the detection and control of risk factors among poor population with exclusive public health coverage and moderate or high CVD risk in Argentina.

The most significant result was observed in the attendance outcome since 82.2% of participants attended at least one clinical visit at the PCC for risk factor control during follow up. The attendance rate far exceeded the level of attendance previously reported by the national program (30%) [9–11] or a prior study (60%) [21] conducted in a similar setting. In addition, the program also improved the attendance to the subsequent clinical visits since the mean number of visits during the study period was 1.8 (SD 0.9), higher than reported by a prior similar cluster trial (1.1) [ 21].

The level of registration in nurses and physicians significantly improved compared to previous reports since 94.1% of physician’s section was completed, BP registration 97.4% (when used to be 43%), and fasting glucose 68.8% (compared to 25.5%) [9–11].

The physicians’ prescription pattern improved throughout the follow-up since they intensified drug treatment in 77% of participants with BP ≥140/90 mmHg and in 79.5% of participants with diabetes. An important cluster trial conducted in Argentina for hypertension control reported 35.5% of drug intensification at 6 months [18]. The performance in prescribing treatments according to GCPs also improved during follow up, especially for statins and antihypertensive drugs. However, it was noted that the adjustment of treatment for diabetes was based mostly on fasting glucose values instead of relying on the suggestion of the Argentinian guidelines of using the value of glycosylated hemoglobin, which is often not available in medical records (18.8%).

During the educational intervention conducted on physicians, we observed that most/many of them were not prescribing drugs for risk factor control at (higher) recommended doses due to an inappropriate fear of adverse effects and the predisposition to accept uncontrolled risk factors. These issues were strongly addressed during the meetings obtaining good results; however, to generate a higher impact on prescriptions patterns, adherence to guidelines and clinical outcomes, it might be necessary to implement the educational outreach visits for a longer time.

On the other hand, physicians conducted several new tasks not performed before, such as evaluation and prescription of statins according to CVD risk and higher medical record registration (notes and drug sections). From our perspective, this intervention pushed for significant improvement of quality of care, despite most physicians not perceiving it as such.

The most significant improvement in effectiveness outcomes was observed among hypertensive participants. Despite the limitations of BP measurements, participants who attended at least one clinical visit lowered mean systolic and diastolic BP values compared to baseline (156.9 to 145.4 mmHg and 92.9 to 88.9 mmHg, respectively) and participants with BP < 140/90 mmHg increased from 20.3 to 35.5% overcoming the historical reference (23.9% [13]). These findings could be mostly related to the improvement of the drug treatment intensification conducted by physicians, although the BP reduction might be also partially due to regression to the mean.

The strengths of this study lie in I) the fact that the intervention was tested in 6 out of 8 PCCs, which makes the results representative of the primary care level of the selected city; II) the demonstration of the potential benefits of including CHWs with a more relevant role in the clinical management of risk factors and a new instrument (CVD form) stimulating teamwork; III) the generation of data to inform the design and implementation of this low-cost intervention at larger scale; IV) the qualitative data analysis allowed to understand this study proposed strategy better, and to consider the positive and negative experiences of the intervention, identify opportunities to improve implementation strategies, and understand the contributions to health care practices.

Some limitations of the study should be mentioned. First, sampling was not at random, and although CHWs were strongly advised against convenience recruitment, selection bias cannot be completely ruled out. If CHWs recruited a higher proportion of people who lived near the PCC or who were more familiar with the PCC, we should expect a less successful attendance rate when the strategy is implemented at a larger scale. Second, clinical measurements data (BP, weight, and height) carry measurement error since were performed using non-standardized procedures. However, random errors do not have any consistent effects across the sample and are very valuable as they describe the daily practice of physicians and nurses. Third, observer bias was also present in the study; it was noted that nurses registered BP measurements rounded to the nearest whole number (i.e. registered 140 mmHg instead of the actual value 138 or 142 mmHg). This error may have led to some degree of misclassification of participants in terms of controlled BP [41]. Finally, the 42.3% of the participants did not answer satisfaction interview. However, the sociodemographic and clinical characteristics between those who answered the interview and those who didn’t are very similar, therefore the selection bias would not significantly affect the results.

## Conclusions

The proposed CHWs-led intervention was feasible and well accepted in improving the detection and treatment of risk factors in the poor population with exclusive public health coverage and moderate or high CVD risk in Argentina. Adding the data collected by CHWs in the community to medical records through the CVD form did not only stimulate teamwork among PCC staff, but it also improved quality of care. Community health workers could have a more relevant role in the detection and clinical management of risk factors in low-income communities.

## Public health implication

The study contributed with the sustainable development goals proposed by the United Nations, which are to reduce premature CVD mortality from non-communicable diseases through prevention and treatment (Goal 3.4) and to achieve universal health coverage, access to quality essential health care services and access to safe, effective, quality and affordable essential medicines for all (Goal 3.8) [42].

This study is also aligned with the efforts made by other organizations to reduce the incidence, morbidity and mortality of CVD worldwide. The World Heart Organization (WHO), in collaboration with the Global Burden of Disease Network and NCD Risk Factor Collaboration (NCD-RisC) derived, calibrated, and validated new cardiovascular risk prediction charts in 21 Global Burden of Disease regions [43]. The HEARTS program provides six technical packages (which includes new cardiovascular risk prediction charts) to be implemented with the aim of improving cardiovascular health, especially in low- and middle-income countries [44].

In the near future, we plan to conduct a cluster randomized trial to test the effectiveness of the proposed intervention at a larger scale using the new risk prediction charts and thereby contribute to the global effort by providing data to update the CVD risk charts developed by the WHO. We will also consider strengthening CHWs and nurses training in the use of the CVD form by increasing the training sessions and prolonging the intervention for at least 18 months. The challenges are to reach similar levels of adoption of the strategy in providers and patients from different settings.

## Supplementary Information

**Additional file 1.**

## Data Availability

The deidentified datasets analyzed in the study reported are available from the corresponding author on reasonable request.

## Abbreviations

CVD: Cardiovascular disease
CHW: Community health workers
PCC: Primary care clinics
GCP: Good clinical practice
EOVs: Educational outreach visits
SD: Standard deviation
IR: Interquartile range
BP: Blood pressure
WHO: World Heart Organization
