# Supplementary material for: An innovative approach to improve the detection and treatment of risk factors in poor urban settings: a feasibility study in Argentina
Source: BMC Public Health. 2021 Mar 22;21:567. doi: 10.1186/s12889-021-10569-3 (PMC7986565; doi:10.1186/s12889-021-10569-3)
Supplement: Supplementary file 1 — Additional file 1. [file 12889_2021_10569_MOESM1_ESM.docx]

**An Innovative Approach to Improve the Detection and Treatment of Risk Factors in Poor Urban Settings: A Feasibility Study in Argentina**

**Authors**

Poggio Rosana^1^ Goodarz Danaei^2^, Laura Gutierrez^1^; Ana Cavallo^1^; María Victoria Lopez^1^; Vilma Irazola^1^

**Affiliation**

1. Department of Research in Chronic Diseases, Institute for Clinical Effectiveness and Health Policy (IECS), Buenos Aires, Argentina

2. Department of Global Health and Populations. [Department of Epidemiology](https://www.hsph.harvard.edu/epidemiology/), Harvard T.H. Chan School of Public Health, Boston, MA, USA

**Correspondence to:**

Rosana Poggio

E-mail: rpoggio@iecs.org.ar. Emilio Ravignani 2024 (C1414CPV), Buenos Aires, Argentina. Phone: + 54-11-4777-8767. Fax: + 54-11-4777-8767-int-27

**Supplementary Figure 1 A: CVD form-side A, CHWs´section**

**Supplementary Figure 1 B: CVD form- side B, Phisicians and nurses sections**

**Supplementary Figure 2: Summary of the study flow**


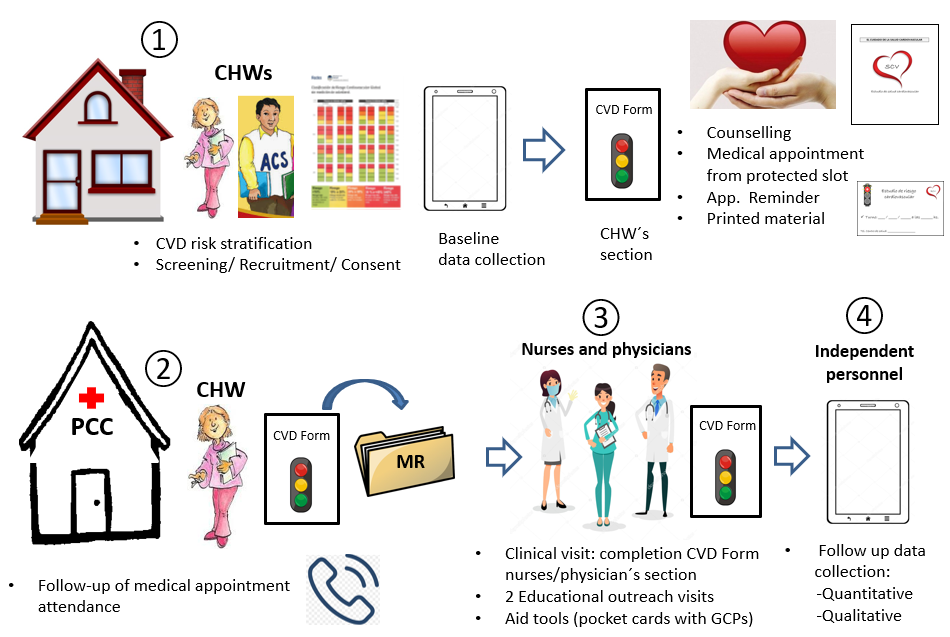


**Supplementary material: Study forms**

**FORM 1: ELEGIBILITY**

1. Identification number
2. Interviewer Code
3. Date of birth
4. Age
5. Sex
6. Is the person prostrate?
7. Are you pregnant?
8. Do you have private or social security health care coverage?
9. Planning to move in the next 6 months?
10. Do you live more than 5 km from the primary care clinic?

**History of cardiovascular disease**

1. Has a doctor ever told you that you had a heart attack or stroke?
2. Has a doctor ever told you that you had a stroke or stroke?
3. Has a doctor ever told you that you had kidney failure or are on dialysis?
4. Have you ever had angioplasty, bypass surgery, or had a stent placed in an artery in your body?

**Risk factors for estimating cardiovascular risk**

1. Has a doctor ever told you that you have diabetes or high blood sugar?
2. Do you currently smoke, or did you stop smoking less than a year ago?

**Blood pressure measurements**

Measure # 1, 2, 3

Systolic pressure (value in mmHg)

Diastolic pressure (value in mmHg)

Average

Systolic pressure (value in mmHg)

Diastolic pressure (value in mmHg)

**Estimated 10 years CVD risk (Result)**

- Low (less than 10%)
- Moderate (10-20%)
- High (20-30%)
- Very high (Greater than 30%)

**Eligibility result**

**If “Eligible”**

1. Do you agree to participate in the study?
2. Have you signed the informed consent documents?

**FORM 2: Baseline data**

**1. Telephone contact**

- Cell phone number
- House phone number
- Current address

**2. What is the highest educational level you have achieved?**

- Did not attend school
- Incomplete primary
- Complete primary
- Incomplete secondary
- Complete secondary
- Incomplete tertiary / university
- Complete tertiary / university
- Do not know / do not want to answer

**3. What is your current employment situation?**

- Worker> 35 hours / week
- Worker ≤ 35 hours / week
- Unemployed and looking for work
- Unemployed and not looking for work
- Retired / pensioner
- In charge of housework
- Other

***4. How many people, including you, live in your household?***

***5. Could you tell us which is the total monthly income of your household?***

*(include income from work, pensions, rents, unemployment insurance, food assistance, etc.)*

- *1 - 10,000 $ARS*
- *20,001- 25,000 $ARS*
- *25,001- 30,000 $ARS*
- *30,001- 35,000 $ARS*
- *35,001 $ARS or more*

***6. How many times have you been to the primary care clinic in the last year to control your risk factors?***

***7. Has a doctor ever told you that you had high blood pressure or hypertension?***

If “Yes”:

7.a. Are you currently taking any drugs to control the pressure?

If “Yes”:

7.b. Type of drug (in milligrams per tablet and tablets per day)

**8. Has a doctor ever told you that you had high cholesterol?**

If “Yes”:

8.a. Are you currently taking any drugs to control cholesterol?

If “Yes”:

8.b Type of drug (in milligrams per tablet and tablets per day)

**9. Has a doctor ever told you that you had high blood sugar or diabetes?**

If “Yes”:

9 a. Are you currently taking any drugs to control diabetes?

If “Yes”:

9.b Type of drug (in milligrams per tablet and tablets per day)

9.c Are you using insulin? (If “Yes”: units per day)

**10. Are you currently taking aspirin daily?**

***FORM 3: Process indicators in household***

**1. Did you conduct a home health promotion session?**

If "YES":

1.a Indicate the modules implemented (you can check more than one option)

- Risk factors and cardiovascular care
- Healthy nutrition
- Active life
- Smoking cessation

1. b How many members of the family group attended the health promotion session? ____

If “NO”:

1.c. Indicate the cause (you can check more than one option)

- Lack of time
- Lack of interest
- Other

**2. Did you provide the medical appointment at the primary care clinic?**

If "NO"

1.a. Indicate the cause

- Lack of time
- Lack of interest
- Other

**FORM 4: Final visit**

***1. At the time of the study final visit, was the participant's medical record present?***

***2. Completeness of the cardiovascular risk form, at the time of the final visit***

a- Community health worker section

- Complete in all sections
- Incomplete in the risk factors section
- Incomplete in the medication section
- Incomplete in both sections
- It was not in the medical record
- Note: __________________________

b. Provider section

- Complete in all sections
- Incomplete in the clinical measurements section
- Incomplete in the previous labs section
- Incomplete in the medication section
- Incomplete in the annotations section
- Incomplete in both sections
- It was not in the medical record
- Note: _______________________

***3. Attendance at the clinic visit If “Yes”***

**-Visits # (1, 2, 3**, **all conducted)**

• Date □ Not available

• Systolic blood pressure: ______ mm Hg □ Not available

• Diastolic blood pressure: ______ mm Hg □ Not available

• Blood glucose value: ______ mg / dL □ Not available

• LDL value: ______ mg / dL □ Not available

**4. Drug treatment (check all that apply, in milligrams per tablet and tablets per day)**

- Aspirin
- Simvastatin
- Enalapril
- Amlodipine
- Hydrochlorothiazide
- Atenolol
- Losartan
- Metformin
- Glibenclamide
- Insulin
- Other

**5. Was lack of adherence to treatment detected?**

**6. Was the laboratory request made?**

**7. Request for a complementary study?**

**FORM 5: LEVEL OF SATISFACION**

*Good morning / afternoon (name of participant)*

*My name is (name of the interviewer) and I am calling to ask you some questions about the visit received in your home on (date of the household visit) conducted by (name of the community health worker) in order to improve cardiovascular health care.*

**1. Was the information provided by the community health worker on CVD health care *totally new*, *somewhat new* or *not at all new*?**

**2. Did you attend the clinical visit at the primary care clinic after the community health worker visited your home?**

**If "Yes":**

2.a. Mode of attendance (READ ALL OPTIONS)

- Used the appointment given by the agent
- You arranged a new appointment on your own
- I attended the clinical visit without an appointment
- Other

**If "NO":**

2.b. Reasons for absence (READ ALL OPTIONS)

- I did not have time
- I could not attend for work reasons
- I Could not attend for lack of money
- I did not want to attend
- I forgot
- I went to the primary care clinic, but the doctor was not available
- I attended the primary care clinic, but the assigned appointment was not respected.
- Other

**3. Please tell us your overall level of satisfaction with the health care received at the primary care clinic. From 1 to 5, where 1 the care was very bad and 5 the care was excellent, what score would you give it?**
